# Astaxanthin Alleviates Chronic Prostatitis *via* the ERK1/2 Signaling Pathway: Evidence from Network Pharmacology and Experimental Validation

**DOI:** 10.2174/0113862073331381240923080152

**Published:** 2024-10-09

**Authors:** Yifu Liu, Liang Huang, Zhicheng Zhang, Qiqi Zhu, Ping Xi, Ting Sun, Binbin Gong

**Affiliations:** 1 Department of Urology, The First Affiliated Hospital, Jiangxi Medical College, Nanchang University, Nanchang, Jiangxi, 330006, China;; 2 Key Laboratory of Urinary System Diseases of Jiangxi Province, Nanchang, Jiangxi, 330006, China;; 3 Department of Urology, The Affiliated Cancer Hospital of Xiangya School of Medicine, Central South University, Hunan Cancer Hospital, Changsha, Hunan, 410013, China

**Keywords:** Astaxanthin, chronic prostatitis, RWPE-1 cells, WPMY-1 cells, molecular docking, inflammation

## Abstract

**Background:**

Astaxanthin (AST) has been widely recognized for its therapeutic potential in chronic inflammatory ailments. This study investigates the therapeutic efficacy and underlying mechanisms of AST in the management of chronic prostatitis (CP).

**Methods:**

Male Sprague-Dawley (SD) rats were randomly divided into control, complete Freund's adjuvant (CFA), and CFA + AST groups. CFA was used to induce the CP model, and saline was used for the control group. Inflammation of the prostate was detected 28 days after oral administration of AST. qRT-PCR and ELISA were used to detect pro-inflammatory factors in RWPE-1 and WPMY-1 cells. Potential targets of AST for CP were explored by network pharmacology, and related proteins were detected by Western blotting.

**Results:**

Oral administration of AST alleviated the increase in prostate stroma and reduced inflammatory cell infiltration in CP rats. The IC_50_ of AST-treated RWPE-1 and WPMY-1 cells for 48 h were 171 and 212.1 μM, respectively. AST pretreatment reduced IL-6 and IL-8 expression in these cells. PPI network, GO, and KEGG enrichment analyses suggested that the anti-inflammatory effect of AST was associated with the ERK1/2 pathway. Western blotting showed that AST inhibited ERK1/2 phosphorylation. In addition, AST and ERK1/2 pathway inhibitors (U0126) synergistically inhibited LPS-induced inflammation in prostate cells.

**Conclusion:**

Our study identified the potential of AST in the treatment of CP. However, subsequent randomized controlled trials are needed to validate its clinical efficacy.

## 1 INTRODUCTION

Prostatitis is a common and heterogeneous disease that seriously affects the quality of life of patients [1]. The prevalence of prostatitis-like symptoms is roughly comparable to that of coronary artery disease and diabetes, with an incidence of approximately 8.2% in the overall population [2]. In the prostatitis syndrome, chronic prostatitis/chronic pelvic pain syndrome (CP/CPPS) accounts for over 90%, undoubtedly being the most common subtype [3]. Patients with CP often suffer from inevitable genitourinary and pelvic pain, lower urinary tract obstruction or irritation, sexual dysfunction, and psychosocial problems [4, 5]. As a result, this syndrome leads to an increasing number of medical visits and a huge economic burden for patients and society.

Understanding of the pathogenesis of CP is still inadequate, and researchers have suggested that the etiology can be categorized as infectious, immunological, neurological, endocrinological, psychological, or a combination of these disciplines [1]. Accordingly, the complexity of etiology has made it difficult for clinicians to find effective treatment strategies for CP. Currently, antibiotics and α-blockers are undoubtedly the most common agents used by clinicians to target CP. However, the efficacy of these agents in patients with CP is limited, especially given the resistance to antibiotics, the risk of hypotension with α-blockers, and conflicting evidence in randomized controlled trials [6-8]. In this context, phytotherapy with fewer side effects is the first consideration for urologists and CP patients. Evidence from randomized controlled clinical trials suggested that the bioflavonoid quercetin and the pollen extract cernilton significantly alleviated clinical symptoms in patients with inflammatory CP and were well tolerated [9, 10]. Undoubtedly, supported by these encouraging results, more superior phytotherapies are to be expected.

Astaxanthin (AST) is a red pigment carotenoid, similar to β-cryptoxanthin, β-carotene, and lycopene, with antioxidant, anti-inflammatory, anti-apoptotic, and immunomodulatory properties [11, 12]. To date, astaxanthin has shown protective potential in a wide range of *in vitro* and *in vivo* studies on human diseases, including diabetes, Parkinson's disease, inflammatory bowel disease, and hepatic and kidney-related disorders [13]. In line with this, a range of clinical evidence further highlights its anti-inflammatory effects in a variety of organs [14-16]. In addition, our previous studies reported that AST can alleviate experimental autoimmune prostatitis by modulating the gut microbiota [17]. Extracellular signal-related kinases 1 and 2 (ERK1/2) are essential components of the mitogen-activated protein kinase (MAPK) phosphorylation cascade and play a central role in many cellular behaviours, including proliferation, differentiation, and migration, and critically shape multiple aspects of the inflammatory response of immune and nonimmune cells [18]. Previous evidence suggested that ERK1/2 was aberrantly activated in a variety of chronic inflammatory diseases, including CP, and that targeted inhibition of the ERK1/2 pathway could reduce the degree of inflammation in various diseases [19-22]. Interestingly, AST has also been shown to alleviate inflammation by inhibiting the phosphorylation of ERK1/2 [23, 24].

However, it remains unknown whether AST has a direct protective effect on inflammatory CP beyond the gut-prostate axis and whether it is related to the ERK1/2 signalling pathway. In this study, we examined the effect of oral AST supplementation on CP rats and validated it *in vitro*. Furthermore, the potential targets of AST for the treatment of CP were analysed using network pharmacology and bioinformatics. This study provides a new perspective on the therapeutic management of CP.

## 2 MATERIALS AND METHODS

### 2.1 Drugs and Materials

AST (≥97% purity, SML0982), LPS (*Escherichia coli* O55: B5, L2880), and complete Freund's adjuvant (CFA; F5881) were purchased from Sigma‒Aldrich (St. Louis, MO, USA). Fetal bovine serum (FBS, 35-081-CV) was obtained from Corning, USA, and DMEM (11960044) was obtained from Gibco (Grand Island, NY, USA). Dimethyl sulfoxide (DMSO, D8371) and PBS (P1020) were purchased from Solarbio (Beijing, China), and TRIzol reagents (ET101-01-V2), EasyScript cDNA kits (EE101-11), and one-step RT‒PCR kits (AE411-02) were purchased from Transgen (Beijing, China). IL-6 (abs510003) and IL-8 (abs510004) ELISA kits were purchased from Absin (Shanghai, China), and CCK8 kits (K1018) were purchased from Apexbio (Houston, TX, USA). The antibodies used in the study are listed below: anti-ERK1/2 (4695T; Cell Signaling Technology, USA), anti-phospho-ERK1/2 (4370T; Cell Signaling Technology, USA), anti-GAPDH (10494-1-AP; Proteintech, China), anti-β-tubulin (10094-1-AP; Proteintech, China), anti-CD3 (17617-1-AP; Proteintech, China), anti-CD45 (60287-1-Ig; Proteintech, China), and secondary antibody (Biotin-conjugated Affinipure Goat Anti-Rabbit IgG, SA00004-2; Proteintech, China). The ERK1/2 inhibitor U0126 (A1337) was purchased from ApexBio (Houston, TX, USA). Penicillin and streptomycin (C100C5) were obtained from NCM Biotech (Suzhou, China). Paraformaldehyde (G1101), ethanol (G1103), hematoxylin-eosin kit (G1076), xylene (G1128), citrate buffer (G1202), horseradish peroxidase (GC305005), bovine serum albumin (GC305010), RIPA lysis buffer (G2002), proteinase inhibitor (G2006), phosphatase inhibitor (G2007), BCA Protein Assay Kit (G2026), SDS-PAGE Rapid Gel Preparation Kit (G2037), TBST buffer (G0004), and PVDF membrane (WGPVDF45) were purchased from Servicebio (Wuhan, China). Colorcode Prestained Protein Marker was purchased from Thermo Scientific (26616; Rockford, IL, USA). Mei5bio (M5-MF212; Beijing, China) and Yeasen (GoldBand Plus 3. 20350ES72; Shanghai, China) were also utilized in this study.

### 2.2 Cell Culture

The human prostate epithelial cell line (RWPE-1, CSTR:19375.09.3101HUMGNHu37) and prostate stromal cell line (WPMY-1, CSTR:19375.09.3101HUMGNHu36) were purchased from the Chinese Academy of Sciences, and both were cultured in DMEM-H. In addition, FBS (10%), penicillin (100 U/mL), and streptomycin (100 µg/mL) were added to the medium.

### 2.3 Cell Viability Assay

AST (5 mg) was added to 1 mL of DMSO for dissolution, and subsequently, 0, 18, 36, 54, 72, 108, and 144 μL were added to 6 ml of DMEM to obtain final concentrations of 0, 25, 50, 75, 100, 150, and 200 μM AST solution, respectively. The number of cells in the 96-well plates was kept at approximately 10^4^ by cell counting, and the cells were treated by adding the different concentrations of AST solutions mentioned above after cell apposition. After 12 h, 24 h, and 48 h, 10 μL CCK-8 solution was added to each well, and the cells were further incubated for 2-4 h. Finally, the absorbance value of each sample was measured at 450 nm.

### 2.4 Quantitative Real-Time PCR

Total RNA was extracted from the NC, AST [75 μM], LPS [2.5 μg/mL], LPS + AST, LPS + U0126 [2 μM], and LPS + U0126 + AST groups, including RWPE-1 and WPMY-1 cells after 2 h of AST/U0126 pretreatment plus 6 h of LPS treatment. cDNA preparation and real-time qPCR were then performed as described in previous studies according to the manufacturer's instructions [25]. β-Actin was used as the internal reference gene, and expression was calculated by the 2^-ΔΔCt^ method. The corresponding primer sequences are mentioned in Table **1**.

### 2.5 Elisa

The levels of the pro-inflammatory factors IL-6 and IL-8 in RWPE-1 and WPMY-1 cell culture supernatant were determined using ELISA kits according to the manufacturer's instructions. Briefly, 100 μL of different concentrations of standards and experimental samples were first added to the corresponding wells and incubated at room temperature for 2 h. The liquid in the wells was then removed, and the wells were washed 3 times with a washing buffer. Afterward, 100 μL of the detection antibody (11 mL of buffer dilution was added to the lyophilized powder of the kit) was added to each well, and the wells were incubated for 2 h at room temperature before being washed again. Next, 100 μL of streptavidin-HRP [1:^40^] was added to the wells, and the wells were incubated for 20 min at room temperature, protected from light, and then washed again. A color developer (100 μL) was then added to each well and incubated for 20 min at room temperature before the reaction was stopped with a termination solution. The wells were zeroed with a blank, the absorbance values were measured at 450 nm, and the concentration of each sample was calculated from the standard curve.

### 2.6 Animals

Male Sprague‒Dawley (SD) rats (200-220 g) were purchased from Hunan SJA Laboratory Animal Co., Ltd. (Changsha, China). The animals were housed in a specific pathogen-free environment at the Animal Centre of the First Affiliated Hospital of Nanchang University at a temperature of 24°C ± 1°C and a relative humidity of 50-60%, where they had free access to food and water.

Eighteen SD rats were randomly assigned to the control, the CFA, and the CFA + AST groups. As shown in Fig. (**1A**), we implemented the induction of the CP Model 1 week after the animals had adapted to their environment, which was based on the description of previous studies [26]. Briefly, after anesthesia, a longitudinal incision of approximately 1 cm was made in the lower abdomen of all rats along the ventral prostate. Subsequently, 100 µL of CFA was injected into each of the left and right lobes of the prostate in the CFA and CFA + AST groups to induce the CP model, while the control group was injected with an equal amount of saline, and the wounds were then closed in layers with absorbable surgical sutures. Gavage of the rats was performed the day after surgery. Based on the experience of previous studies, the dose of AST was determined to be 100 mg/kg/d dissolved in 2 mL of olive oil administered by gavage to rats in the CFA + AST group, while rats in the control and CFA groups were given equal amounts of olive oil [27, 28]. This operation was performed once a day until day 28, when the rats were euthanized by inhalation of an excessive amount of isoflurane. Half of the prostate tissue obtained after the dissection of the rats was used for histological evaluation and the other half was used for qRT-PCR and Western blotting.

### 2.7 Prostate Index

At the end of four weeks, all rats were weighed and then sacrificed. The weight of the prostate was determined using an accurate electronic balance.

Prostate index = [prostate weight/rat weight] × 1000 [29].

### 2.8 Hematoxylin and Eosin Staining

The prostate tissue was fixed in 4% paraformaldehyde for approximately 48 h, followed by ethanol dehydration, paraffin embedding, and sectioning (5 μm). Next, hematoxylin and eosin (H&E) staining was performed sequentially. Finally, the tissue sections were observed by electron microscopy, and the histological scoring criteria were as described in previous studies [26].

### 2.9 Immunohistochemical Staining

Paraffin sections were dewaxed with xylene and then hydrated with various concentrations of alcohol, followed by antigen repair using citrate buffer (pH 6.0) at 95°C. Next, the slides were incubated with 3% H_2_O_2_ solution for 10 min at room temperature and blocked with 5% bovine serum albumin. The slides were then incubated with primary antibody ([1:500] CD3, [1:4000] CD45) at 4°C overnight. After 3-5 washes with PBS, the slides and the secondary antibody [1:500] coupled to horseradish peroxidase were incubated at 37°C for 1 h. Diaminobenzidine was used to detect the immune signal.

### 2.10 Identification of AST-Related Targets

AST-related targets in Homo sapiens were identified through the following public databases: Bioinformatics Analysis Tool for Molecular MechANism database [http://bionet.ncpsb.org/batman-tcm/index.php/home] [30], Comparative Toxicogenomics database [http://ctdbase.org] [31], PharmMapper [http://www.lilab-ecust.cn/pharmmapper] [32], Similarity Ensemble Approach [https://sea16.docking.org/] [33], and Swiss Target Prediction [http://swisstargetprediction.ch/] databases [34]. After removing duplicate genes from these six datasets, the pooled molecules were used as candidate genes associated with AST.

### 2.11 CP-Related Target Prediction Based on GeneCards

GeneCards [https://www.genecards.org/] automatically integrates gene-centric data from approximately 150 web resources, including genomic, transcriptomic, proteomic, genetic, clinical, and functional information [35]. Using this database, we screened for target genes associated with "chronic prostatitis", of which relevance scores greater than 10 were included in this study [36].

### 2.12 Construction of PPI Networks and Enrichment Analysis

Common targets of AST and CP were identified by the Venn diagram. After obtaining the common targets, a PPI network was constructed with the help of the STRING database [https://cn.string-db.org/], with the minimum required interaction score threshold set at 0.900 [37, 38]. Subsequently, the initial version of the PPI network was imported into Cytoscape (version 3.9.1). GO functional and KEGG pathway enrichment analyses were performed on common targets of AST and CP to explore potential biological functions and key targets; these were implemented through the R package "ClusterProfiler".

### 2.13 Molecular Docking

The 3D coordinates of ERK1 [MAPK3; PDB ID: 2ZOQ] and ERK2 [MAPK1; PDB ID: 6GJB] were obtained from the RCSB protein data bank [https://www.rcsb.org/] and the 3D structures of AST and SCH772984 were obtained from PubChem [https://pubchem.ncbi.nlm.nih.gov]. Mcule.com [https://mcule.com] is an online drug discovery platform that provides high-quality molecular modelling tools. With the help of this platform, we imported the 3D coordinates of the molecules and the 3D structure of the drug for molecular docking. Discovery Studio software was then used to visualize the molecular docking results after they were exported from the platform. In general, a binding free energy value of less than -4.25 kcal/mol, -5.0 kcal/mol, or -7.0 kcal/mol indicates certain, good, or strong binding activity between the ligand and the receptor, respectively [39, 40].

### 2.14 Western Blotting

Western blotting was performed in detail as described previously [41]. Briefly, RWPE-1 and WPMY-1 cells were seeded in 6-well plates and treated with LPS [2.5 μg/mL] with or without the addition of AST [75 μM]/U0126 [2 μM] in advance for 2 h. Cells were subsequently collected and lysed by adding RIPA lysis buffer, protease inhibitor, and phosphatase inhibitor for 30 min at 4°C. If the sample was prostate tissue, a homogenate was made by adding the same reagents as above, followed by lysis at 4°C. After centrifugation at 12000 rpm for 20 min, the supernatant was collected, and the protein was quantified using the BCA Protein Assay Kit. Afterward, the proteins were added to a 10% SDS‒PAGE gel for electrophoresis and then transferred to a PVDF membrane. After blocking with 5% bovine serum albumin for 1h at room temperature, incubate simultaneously overnight at 4°C with the corresponding primary antibody (anti-ERK1/2, 1:1000; anti-phospho-ERK1/2, 1:2000; anti-GAPDH, 1:5000; anti-β-tubulin; 1:2000). After washing with TBST, the membranes were incubated with the secondary antibody [1:5000] for 1 h at room temperature. Enhanced chemiluminescence (ECL) detection was used to visualize the target proteins, and ImageJ software was used to quantify the protein bands.

### 2.15 Statistical Analyses

To verify the reliability of the results, each experiment was performed at least three times independently, and the data are expressed as the mean ± SD. Data were analysed using *t-*tests, one-way analysis of variance [ANOVA], and Bonferroni *post hoc* tests based on GraphPad Prism 8.0 software. Differences between groups were considered statistically significant at a *P* value of < 0.05.

## 3 RESULTS

### 3.1 AST Alleviates Prostate Inflammation in the CP Rat Model

The prostate index is usually used to characterize the degree of inflammation in chronic prostatitis [42]. We observed an increase in prostate volume and prostate index in a model of CP induced by CFA injection, and this pathological change was alleviated to some extent by supplementation with AST (Fig. **1B**-**C**). As shown in HE staining (Fig. **1D**), after 28 days of constructing the CP rat model, the stromal component of the prostate gland was significantly increased, accompanied by a large infiltration of inflammatory cells and different degrees of glandular damage. Interestingly, oral administration of AST alleviated these histological changes in CP model rats. CD3 and CD45 were used as biomarkers for T cells and leukocytes, respectively, and these immune cells were significantly increased in the CP model [43]. Indeed, our results confirm this, with a significant increase in CD3+ T cells and CD45+ leukocytes in the prostate of CFA-induced CP model rats. Additionally, it is noteworthy that AST treatment significantly inhibited the infiltration of these immune cells (Fig. **1E**). Furthermore, IL-6, IL-8, and IL-1β have been identified as key inflammatory mediators of CP in previous studies [44-46]. We verified the expression of inflammatory factors in prostate tissue samples. As the base sequence of the IL-8 gene is not known in rats, we examined the expression of its main receptor, CXCR2 [47]. Consistent with the histological phenotype, treatment with AST significantly reduced the upregulation of mRNA expression of IL-6, CXCR2, and IL-1β in the CFA-induced CP model. These results suggest that oral supplementation of AST is effective in alleviating prostate inflammation in a CFA-induced CP rat model accompanied by downregulation of the pro-inflammatory factor IL-6, CXCR2, and IL-1β.

### 3.2 AST Alleviates LPS-induced Upregulation of Pro-inflammatory Cytokines in Prostate Cells

First, to determine the maximum safe concentration of AST, we performed a cell viability assay using RWPE-1 and WPMY-1 cells. As shown in Fig. (**2A**), different concentrations of AST were used to treat RWPE-1 and WPMY-1 cells, and a concentration-dependent decrease in cell viability was observed when AST concentrations were greater than 75 μM. Furthermore, when RWPE-1 and WPMY-1 cells were treated with AST for 48 h, the IC_50_ was 171 μM and 212.1 μM, respectively. Therefore, 75 μM of AST solution was determined to be a safe dose and was used for subsequent experiments. Both mRNA and protein expression of IL-6 and IL-8 were significantly up-regulated after 6 h of LPS treatment of RWPE-1 and WPMY-1 (Fig. **2B**-**C**). However, this was effectively suppressed by AST pretreatment (Fig. **2B**-**C**).

### 3.3 Prediction of Relevant Targets

Given that AST has shown benefits for CP in both *in vivo* and *in* *vitro* experiments, we then explored its potential mechanisms. By utilizing public databases, we predicted the relevant targets for AST and CP. As shown in Fig. **3A**, 462 AST-related targets were obtained after screening, while 817 potential targets for CP were predicted. Subsequently, we performed a cross-tabulation analysis of the targets in these two parts and obtained 138 overlapping molecules (Fig. **3B**). Based on these results, PPI networks were constructed to identify the hub genes in AST and CP (Fig. **3C**). MAPK1, MAPK3, and TP53 were identified as the most critical targets in this network.

### 3.4 GO and KEGG Enrichment Analyses

Using the 138 overlapping genes described above, we performed GO and KEGG enrichment analyses. In biological processes, "gland development, responses to oxidative stress, positive regulation of MAPK cascade, responses to lipopolysaccharides, positive regulation of cytokine production and regulation of inflammatory response" were significantly enriched (Fig. **4A**). In the cell component category, these genes were mainly localized in the vesicle lumen and membrane regions (Fig. **4B**). In contrast, in terms of molecular function, these overlapping molecules may have an impact, including signaling receptor activator activity, receptor-ligand activity, cytokine receptor binding, cytokine activity, *etc*. (Fig. **4C**). The results of the KEGG enrichment analysis suggested that the MAPK signaling pathway might be a potential pathway for the role of AST in CP (Fig. **4D**). It is well known that crosstalk between pro-inflammatory cytokines and related signaling pathways together promotes the body's inflammatory cascade response [48, 49]. As the MAPK signaling pathway was significantly enriched in both GO analysis and KEGG analyses, coupled with the fact that MAPK1 [ERK^2^] and MAPK3 [ERK^1^] are key components of the ERK1/2 signaling pathway, we hypothesize that the protective effect of AST on CP may be related to the ERK1/2 signaling pathway.

### 3.5 Molecular Docking Suggests that AST Can Bind Tightly to ERK1/2

The above results suggest that the anti-prostatitis effect of AST is related to the activation status of the ERK1/2 pathway, but how AST affects the ERK1/2 pathway remains unclear. Considering that ERK1 and ERK2 are predicted to be key genes in AST for CP, we performed molecular docking to examine the binding ability of AST to these two targets. As shown in Fig. (**5A**), AST can interact with Glu267 and His195 in ERK1 via 1 hydrogen bond and with Leu355, Pro356, and His195 in ERK1 via an alkyl group. Alternatively, the structure of AST can interact with Pro229, Leu278, Leu241, Lys270, Ile227, and Ile243 in ERK2 via an alkyl bond, with Pro268 and Leu267 via 2 alkyl bonds, and with His239 in a Pi-Pi stacking interaction.

Binding energy calculations are often used to evaluate the degree of affinity of a molecule for its target; the lower the binding energy, the higher the affinity of the receptor for the ligand and the more stable the conformation [40]. Our results showed that the binding energies of AST to ERK1 and ERK2 were -6.01 and -8.034 kcal/mol, respectively. To better evaluate the binding ability of AST to ERK1/ERK2, we used SCH772984, a highly selective ERK1/2 inhibitor, as a control group. As shown in Fig. (**5B**), we performed a molecular docking analysis of SCH772984 and ERK1/ERK2 with binding energies of -8.643 and -9.05 kcal/mol, respectively. Taken together, the above evidence suggests that AST stably binds ERK1 and ERK2, which may be the structural basis for AST to regulate phosphorylation of the ERK1/2 signaling pathway.

### 3.6 AST Inhibits Activation of the ERK1/2 Pathway In Vivo and In Vitro

We next investigated the activation status of the ERK1/2 pathway *in vivo*. As expected, the ERK1/2 pathway was significantly activated in the CP rats, and AST administration was also effective in preventing this phenotype (Fig. **6A**). Furthermore, we observed that phosphorylation levels of ERK1/2 were significantly upregulated in RWPE-1 and WPMY-1 cells after 2 h of LPS treatment, whereas AST pretreatment significantly inhibited the activation of this pathway (Fig. **6B** and **C**). Thus, based on this evidence, we infer that the ERK1/2 signaling pathway may be involved in the anti-inflammatory role of AST in CP.

### 3.7 AST and ERK1/2 Pathway Inhibitor U0126 Synergistically Inhibit LPS-induced Inflammation in Prostate Cells

To further elucidate the ERK1/2 signaling pathway in relation to the anti-inflammatory effects of AST, we investigated the effect of U0126, a classical inhibitor of the ERK1/2 signaling pathway, on the inflammatory response of LPS-treated RWPE-1 and WPMY-1 cells. As shown in Fig. (**7A**), treatment with LPS resulted in increased ERK1/2 phosphorylation in RWPE-1 and WPMY-1 cells, whereas pretreatment with U0126 attenuated this alteration. Furthermore, pretreatment of LPS-induced prostate cells with U0126 plus AST further enhanced the inhibitory effect of U0126 on ERK1/2 phosphorylation (Fig. **7A**). Consistent with this, the mRNA levels of IL-6 and IL-8 were also further reduced in the LPS + U0126 + AST group compared to the LPS + U0126 group (Fig. **7B**). Thus, these results not only reveal that AST enhances the effect of U0126 on CP but also further support that the ERK1/2 pathway is involved in the anti-prostatitis effect of AST.

## 4 DISCUSSION

CP has been a focus of urologists and researchers for a long time, with many recent studies shifting towards investigating chronic inflammatory and immunological mechanisms. These studies primarily concentrate on the role of immune cells, oxidative stress, and inflammatory factors, whether acting alone or in combination [26, 43, 50-53]. Among the therapeutic strategies, antioxidants or phytotherapy are emerging as promising options [54-56]. AST, as an approved dietary supplement, has been found to alleviate a variety of inflammatory diseases, including neuroinflammation [57], liver inflammation [58], kidney inflammation [59], digestive tract inflammation [60], and vascular inflammation [61]. In this study, we demonstrated that AST effectively alleviates inflammation in prostate cells and tissues.

Inflammatory processes are often intricately regulated by signals involved in the initiation, maintenance, and shutdown of inflammation, and an imbalance between these signals allows the inflammatory response to spiral out of control, ultimately leading to cell and tissue damage [62]. During this process, T cells and macrophages not only play a role in antigen presentation and phagocytosis but also participate in immune regulation through the production of various inflammatory factors. In addition, the role of nonimmune cells in the inflammatory process cannot be overlooked, especially considering the position of intestinal epithelial cells and intestinal fibroblasts in inflammatory bowel disease [63]. Here, we demonstrated that AST effectively inhibits the secretion of IL-6 and IL-8, inflammatory cytokines that have been considered classical biomarkers of CP, in RWPE-1 and WPMY-1 cells [46]. However, the inflammatory inhibitory effect of AST on RAW264.7 macrophages *in vitro* has been repeatedly demonstrated in previous studies [23, 64]. Equally importantly, we observed that AST effectively inhibited the aggregation of CD3+ T cells and CD45+ leukocytes around the prostate alveoli in a CP rat model. Therefore, it is reasonable to assume that the anti-inflammatory effect of AST in CP may be related to its synergistic effect on immune and nonimmune cells.

The development of network pharmacology has facilitated the study of "drug-target-disease" and is expected to provide strong support for the future of precision drug therapy [65, 66]. Indeed, previous studies reported that the anti-inflammatory mechanisms of AST are complex and extensive [13]. Through the use of network pharmacology methods, we constructed a PPI network and identified MAPK1 (ERK2) and MAPK3 (ERK1) as key targets for the treatment of CP with AST. Furthermore, GO and KEGG analyses showed that the MAPK signaling pathway was significantly enriched. Thus, the ERK1/2 signaling pathway became the focus of our study. GO analysis predicted the potential anti-inflammatory and antioxidant effects of AST, providing theoretical support for the therapeutic potential of AST in CP. Taken together, the network pharmacology approach provides ideas for the mechanistic study of AST in CP.

MAPKs are classical signaling transducers characterized as mediators of acute and chronic inflammation [67]. Among them, ERK1/2 is one of the main members of the MAPK family. In general, activation of the ERK1/2 pathway is dependent on the stimulation of inflammatory factors and oxidative stress, and in turn, activation of the pathway can further amplify inflammation, forming a loop. Notably, the ERK1/2 pathway has been found to be significantly activated in CP models, including the CFA-induced rat model and the experimental autoimmune prostatitis model [22, 53]. Consistent with these studies, the ERK1/2 signaling pathway could be activated in both the cellular and animal models established in this study. However, again, this effect could be downregulated in both models by AST administration. It is well known that *in vitro* experiments, phosphorylation of proteins occurs relatively quickly and tends to peak rapidly, whereas peak secretion of inflammatory factors takes much longer [68]. Therefore, in this study, we chose two-time points, 2 h and 6 h of LPS treatment, to detect ERK1/2 phosphorylation and inflammatory factor secretion, respectively.

Furthermore, in cellular models, the ERK1/2 inhibitor U0126 was shown to alleviate LPS-induced upregulation of pro-inflammatory cytokines, and this effect could be enhanced by pretreatment with AST. Clearly, this evidence supports our inference that the protective effect of AST on CP is at least partially dependent on the regulation of the ERK1/2 signaling pathway. However, the synergistic effect of AST with U0126 may indicate a difference in the anti-prostatitis mechanism of these two drugs. For example, U0126 inhibits the ERK1/2 pathway by selectively inhibiting MEK1/2, whereas the molecular docking evidence from this study suggests that AST may produce this effect by binding to ERK1 and ERK2. In addition, AST may affect the inflammatory response of the prostate by acting in conjunction with other pathways. After all, phosphorylation of P38, JNK, and NF-κB can be enhanced in prostatitis, and the association of AST with these pathways has been demonstrated in multiple disease models [13, 22].

Overall, the above evidence provides preliminary confirmation that AST may be an effective treatment option for CP. However, there are still some drawbacks that should not be overlooked. First, considering the basic principles of animal welfare, the presence or absence of effects of AST on control rats was not explored, and only 100 mg/kg/d AST was selected for *in vivo* studies. Second, AST has long been considered an antioxidant, and GO analysis in this study showed that AST is associated with oxidative stress in CP. Therefore, it cannot be ruled out that AST alleviates prostatitis through antioxidative stress. Thirdly, ERK1 and ERK2 act as the final link in the ERK1/2 signaling pathway, which can influence the inflammatory cascade response by targeting multiple downstream genes. However, these detailed mechanisms are still unclear in CP, and subsequent studies need to be conducted in this direction. Finally, although molecular docking has shown that AST has good affinity for ERK1 and ERK2, it remains unclear whether AST affects phosphorylation of the ERK1/2 pathway by binding directly to ERK1 and ERK2, and knockout experiments on key targets are needed in subsequent work to confirm this finding.

## CONCLUSION

Our results showed that AST inhibits the inflammatory response of prostate cells and reduces the infiltration of immune cells in the rat prostate tissue. The mechanism may involve inhibition of the activation of the ERK1/2 signaling pathway. Therefore, subsequent randomized controlled trials need to be performed to validate its clinical efficacy in patients with CP.

## AUTHORS’ CONTRIBUTIONS

YL and LH wrote the manuscript. ZZ, QZ and PX took part in the data collection. Ting Sun and Binbin Gong contributed to the concept or design of the study. All authors reviewed the results and approved the final version of the manuscript.

## Figures and Tables

**Fig. (1):**
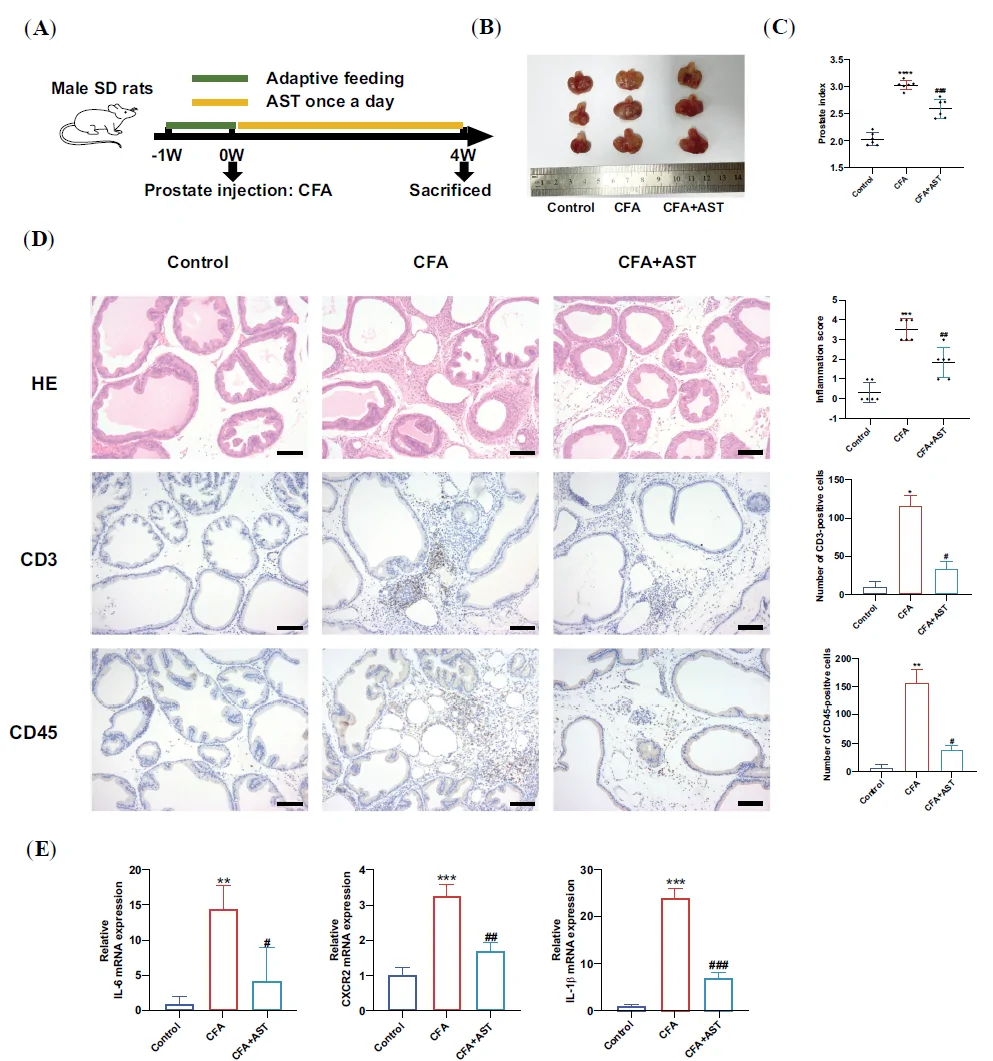
AST alleviates prostate inflammation in a rat CP model. (**A**) Experimental protocol. (**B**) Morphological changes in the prostate. (**C**) AST relieves the increased prostate index caused by CP. (**D**) AST alleviates CFA-induced histological destruction of the prostate and infiltration of CD3+ lymphocytes and CD45+ lymphocytes (bar = 100 μm). (**E**) AST inhibits the mRNA upregulation of IL-6, CXCR2, and IL-1β in prostate tissue of the CP model. Values are displayed as the mean ± SD (n = 3-6). Significant differences are presented as *P < 0.05, **P < 0.01, ***P < 0.001, and ****P < 0.0001 in comparison to the control group. #*P* < 0.05, ##*P* < 0.01 and ###*P* < 0.001, in comparison to the CFA group. AST, astaxanthin. CP, Chronic prostatitis. CFA, Complete Freund's adjuvant.

**Fig. (2):**
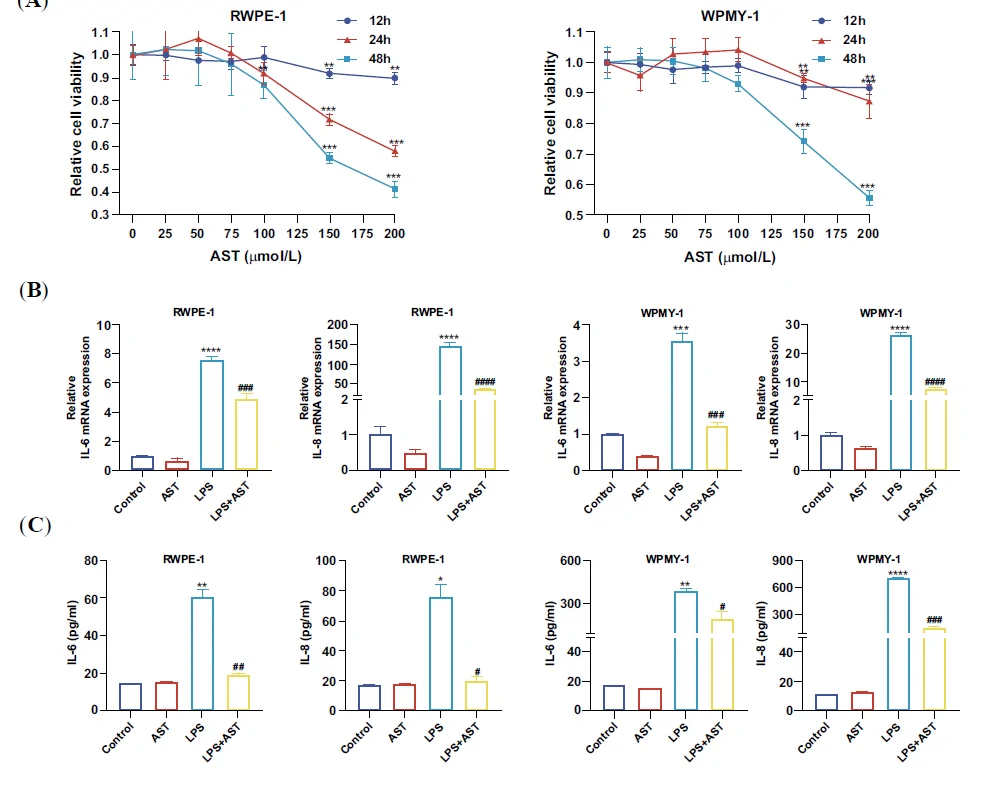
AST reduces the LPS-induced elevation of pro-inflammatory cytokines. (**A**) Safe dose determination of AST in RWPE-1 and WPMY-1 cells. (**B**) AST alleviates LPS-induced mRNA upregulation of IL-6 and IL-8 in RWPE-1 and WPMY-1 cells. (**C**) AST alleviates LPS-induced upregulation of the protein levels of IL-6 and IL-8 in RWPE-1 and WPMY-1 cells. Values are displayed as the mean ± SD (n = 3). Significant differences are presented as *P < 0.05, **P < 0.01, ***P < 0.001, and ****P < 0.0001 in comparison to the control group. #*P* < 0.05, ##*P* < 0.01, ###*P* < 0.001 and ####*P* < 0.0001, in comparison to the LPS group. AST, astaxanthin.

**Fig. (3):**
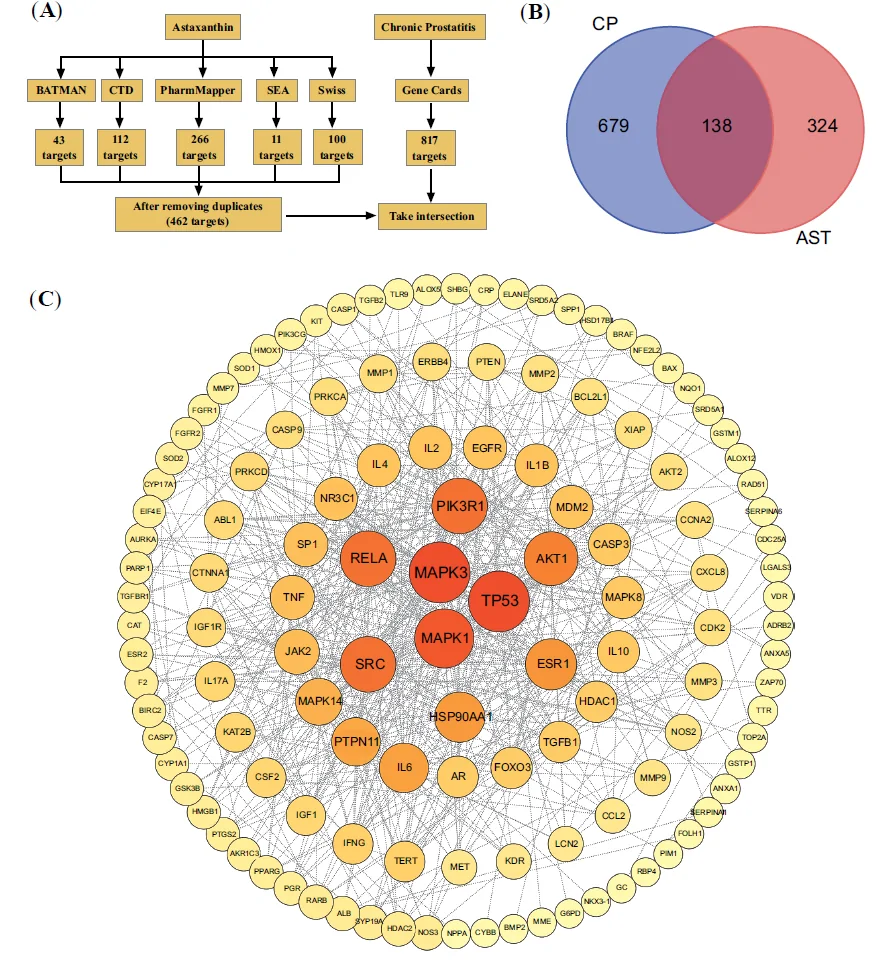
Target identification and PPI network construction. (**A**) Target screening process for AST and CP. (**B**) Venn diagram of AST-related targets and targets of CP. (**C**) PPI network for AST and CP common targets (each node represents a protein target, and the edges represent protein-protein associations). **Abbreviations:** BATMAN, Bioinformatics Analysis Tool for Molecular MechANism; CTD, Comparative Toxicogenomics database; SEA, Similarity Ensemble Approach; Swiss, SwissTargetPrediction databases; PPI, Protein-protein interaction; AST, Astaxanthin; CP, chronic prostatitis.

**Fig. (4):**
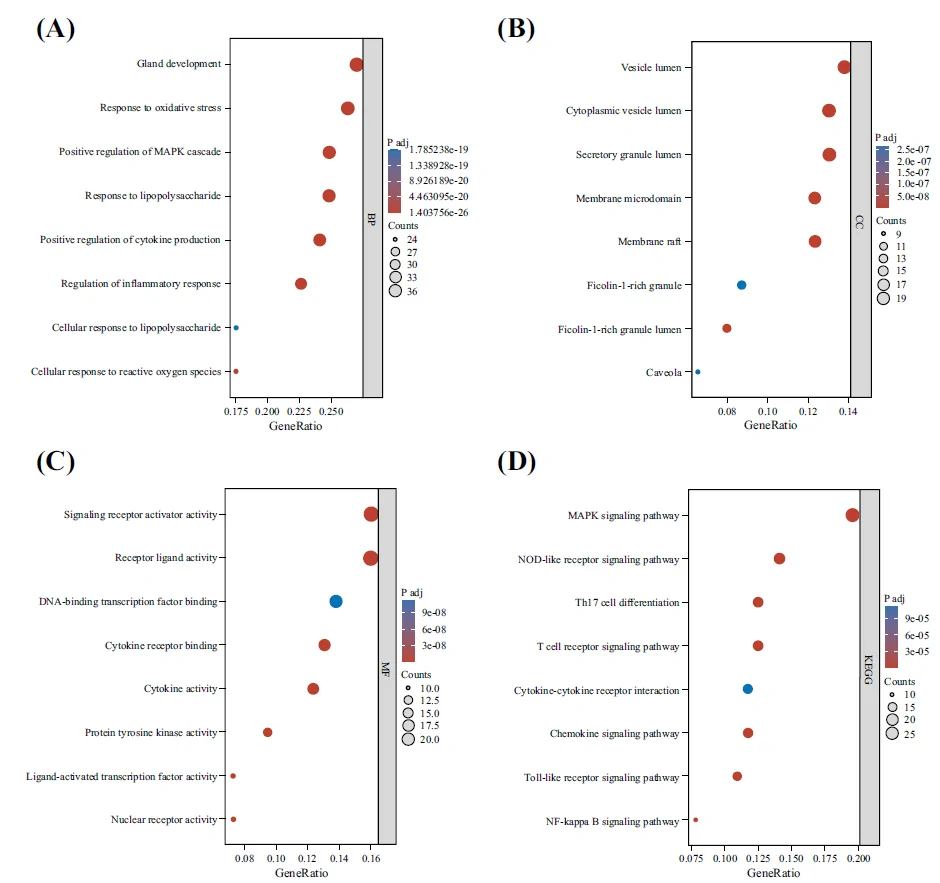
GO and KEGG enrichment analyses based on common targets of CP and AST. (**A**) BP. (**B**) CC. (**C**) MF. (**D**) KEGG enrichment analysis. **Abbreviations:** AST, astaxanthin; CP, chronic prostatitis; GO, gene ontology; KEGG, Kyoto Encyclopedia of Genes and Genomes; BP, biological processes; CC, cell component; MF, molecular function.

**Fig. (5):**
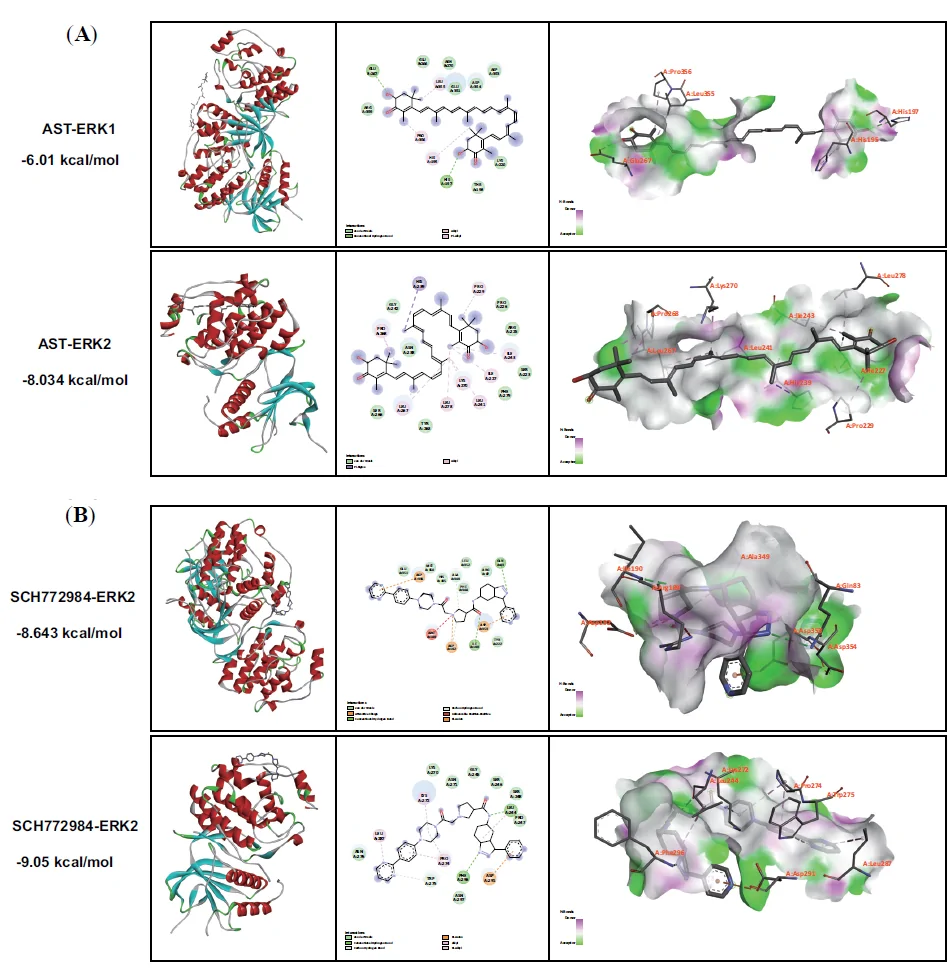
Molecular docking results of AST and SCH772984 with ERK1/2. (**A**) Docking model of AST with ERK1/2. (**B**) Docking model of SCH772984 with ERK1/2. From left to right: 3D, 2D, and spatial structures. AST, astaxanthin.

**Fig. (6):**
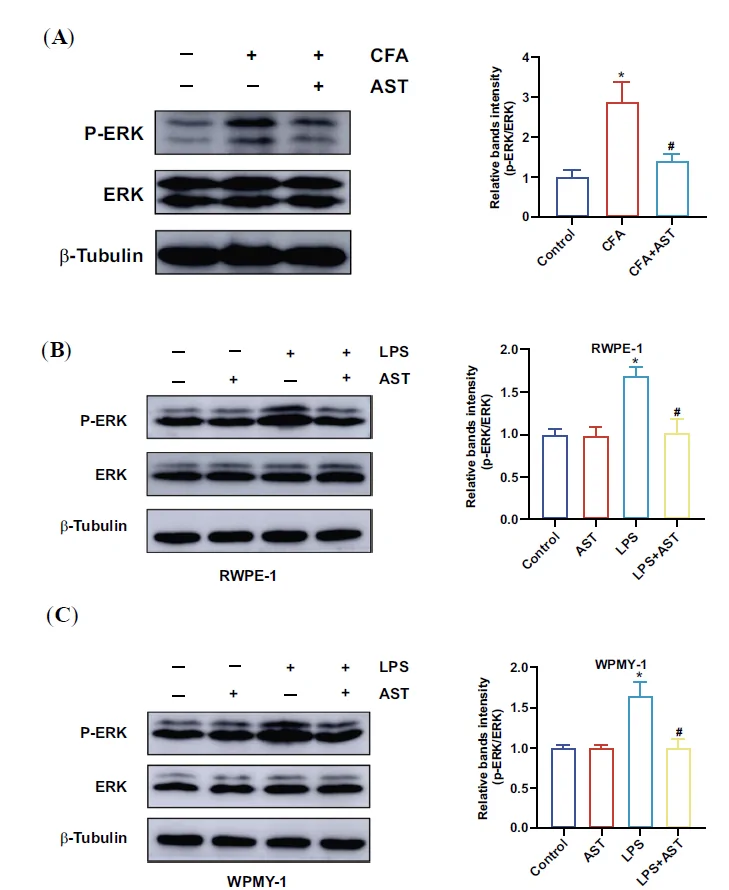
AST inhibits activation of the ERK1/2 pathway *in vivo* and *in vitro*. (**A**) AST downregulates CFA-induced ERK1/2 phosphorylation in prostate tissue. (**B-C**) AST alleviates LPS-induced ERK1/2 phosphorylation in RWPE-1 and WPMY-1 cells. Values are displayed as the mean ± SD [n = 3]. Significant differences are presented as *P < 0.05 in comparison to the control group. #*P* < 0.05, in comparison to the LPS group. AST, astaxanthin. CFA, Complete Freund's adjuvant.

**Fig. (7):**
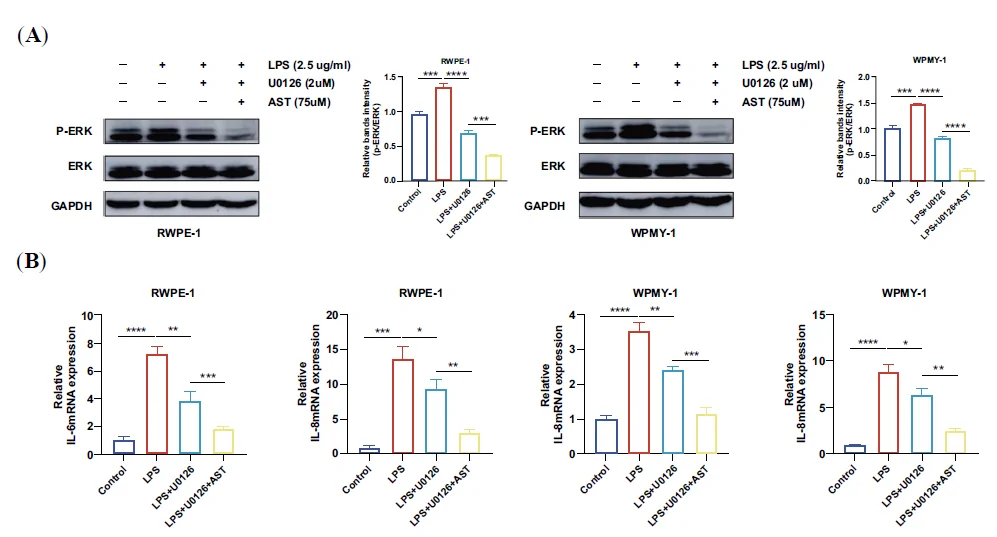
AST enhances the anti-inflammatory effects of U0126. (**A**) AST enhances the inhibitory effect of U0126 on LPS-induced ERK1/2 phosphorylation. (**B**) AST further attenuates the LPS-induced upregulation of IL-6 and IL-8 mRNA levels on the basis of U0126. Values are displayed as the mean ± SD (n = 3). Significant differences are presented as *P < 0.05, **P < 0.01, ***P < 0.001 and ****P < 0.0001. AST, astaxanthin.

**Table 1:** Primer sequences.

**Gene**	**Species**	**Sequence**
IL6	Human	Forward:CCTTCTCCACAAACATGTAACAAGA Reverse:CACCAGGCAAGTCTCCTCATT
IL8	Human	Forward:GACATACTCCAAACCTTTCCA Reverse:AACTTCTCCACAACCCTCTG
β-actin	Human	Forward:TCTCCCAAGTCCACACAGG Reverse:GGCACGAAGGCTCATCA
IL6	Rat	Forward:CACTTCACAAGTCGGAGGCT Reverse:TCTGACAGTGCATCATCGCT
CXCR2	Rat	Forward:CAGAGACTTGGGAGCCACTC Reverse:GCTGAAATTATCCACCCTGATT
IL1β	Rat	Forward:TGGCAACTGTCCCTGAACTC Reverse:CCCAAGTCAAGGGCTTGGAA
β-actin	Rat	Forward:CCTCTGAACCCTAAGGCCAA Reverse:AGCCTGGATGGCTACGTACA

## Data Availability

The data that support the findings of this study are available from the corresponding authors upon reasonable request. The manuscript data were obtained from the following public databases: Molecular MechANism database [http://bionet.ncpsb.org/batman-tcm/index.php/home], Comparative Toxicogenomics database [http://ctdbase.org/], PharmMapper [http://www.lilab-ecust.cn/pharmmapper], Similarity Ensemble Approach [https://sea16.docking.org/], SwissTargetPrediction databases [http://swisstargetprediction.ch/], GeneCards [https://www.genecards.org/], STRING database [https://cn.string-db.org/], RCSB protein data bank [https://www.rcsb.org/], PubChem [https://pubchem.ncbi.nlm.nih.gov], and Mcule.com [https://mcule.com].

## LIST OF ABBREVIATIONS

AST: Astaxanthin
CP: Chronic Prostatitis
ECL: Enhanced Chemiluminescence
MAPK: Mitogen-activated Protein Kinase
